# Protecting reproductive rights in an era of declining fertility: charting a path for the SRHR community

**DOI:** 10.3389/fgwh.2026.1952617

**Published:** 2026-09-28

**Authors:** Danielle Garfinkel, Kaitlyn Patierno, Jennifer Sciubba, Gizachew Balew Jenbere, Moke Magoma, Traci L. Baird

**Affiliations:** 1 EngenderHealth, Washington, DC, United States; 2 Population Reference Bureau, Washington, DC, United States; 3 EngenderHealth, Addis Ababa, Ethiopia; 4 EngenderHealth, Dar Es Salaam, Tanzania

**Keywords:** fertility decline, informed choice, low- and middle-income countries, reproductive autonomy, sexual and reproductive health and rights (SRHR), sub-Saharan Africa

## Abstract

Efforts to protect and expand sexual and reproductive health and rights (SRHR) are at a critical juncture. In many countries, concerns about declining fertility are increasingly shaping development priorities, raising the risk that demographic and socio-economic anxieties overshadow rights and informed choice. While growing attention to fertility decline does not inherently threaten SRHR, alarmist narratives can reshape public discourse, service and financing priorities, and policy in ways that constrain reproductive autonomy. This is of particular concern in low- and middle-income countries, where fertility trends vary widely, narratives can be applied without context, and evidence to guide rights-based approaches remains scarce. Early interviews and consultations with SRHR stakeholders in four sub-Saharan African countries and with global leaders suggest that concerns about declining fertility are not yet widely reflected in policy reversals, but that such changes may be on the horizon and stakeholders lack the information and tools needed for tailored communication and engagement strategies. Without a proactive, evidence- and rights-based response, SRHR efforts risk losing support at a time when women’s and girls’ needs remain urgent. Advocates, policymakers, and practitioners have a window of opportunity to elevate the issue and identify effective strategies to protect SRHR in the context of fertility decline. Doing so requires a focus on locally relevant evidence, rights-affirming communications, protection of services and financing, and policy monitoring and safeguarding.

## Introduction

Global fertility rates are declining, with two-thirds of the world's population living in places where the total fertility rate (TFR) is below the typical replacement level of 2.1 children per woman (1). Governments and other stakeholders are grappling with the economic implications of this shift. Fertility alarmism has gone global, with below-replacement fertility portrayed not just as a cautionary tale, but as an outright crisis (2). These narratives have quickly taken hold across Europe, Asia, South America, and the U.S., influencing policy in ways that threaten individual rights and family choice (3, 4).

In low- and middle-income countries (LMICs), fertility levels and trajectories vary widely and remain relatively high, especially across Africa. How far concerns about declining fertility have begun to shape policy and public discourse in these contexts remains unclear. Yet global trends suggest that such concerns—and the alarmist narratives that can accompany them—are likely to emerge well before fertility reaches very low levels, with potentially significant implications for sexual and reproductive health and rights (SRHR), particularly as funding for SRHR declines following substantial cuts to USAID programs and their support for training, services and supplies for family planning (FP). Without careful attention, hard-won progress could be vulnerable to erosion.

Fertility decline itself is not a threat to SRHR, but risks emerge when raising birth rates becomes a policy objective that overrides reproductive autonomy and shapes health priorities. Increased attention to fertility rates has coincided with a rising pronatalist movement, that could promote policies prioritizing demographic targets over rights. Anti-rights actors may leverage alarmist rhetoric to advance agendas that constrain individual and family choice, endangering the rights-based, people-centered commitments of the ICPD Programme of Action (PoA) (5). Unchecked, these dynamics may reshape narratives and financing and service delivery commitments, and support policies that constrain choice and erode rights. The global SRHR community can learn across regions and develop strategies that safeguard choice before fertility decline discourse gains traction.

SRHR stakeholders have a unique opportunity—and responsibility—to prepare for fertility decline before crisis-based narratives take hold. **This commentary outlines that preparation:** stronger evidence, rights-affirming narratives, protection of health services and resilient financing, and vigilance as policy and public discourse evolve.

We explored prevailing perceptions and issues driving fertility decline concerns expressed or encountered by SRHR colleagues. Our perspective is informed by informal interviews with SRHR stakeholders in Côte d'Ivoire (*n* = 11), Ethiopia (*n* = 8), Nigeria (*n* = 9), and Tanzania (*n* = 8), including representatives from government, civil society organizations, health facilities, population and statistics agencies, and research institutions, as well as small group consultations with twelve global SRHR leaders. The interviews and consultations reveal an evolving landscape, with views varying across settings (6).

Drawing on these conversations and global patterns, we call on the SRHR community to anticipate emerging risks and engage proactively and strategically on fertility decline across national and global platforms.

## Early insights and open questions on fertility decline and SRHR

Despite global attention to fertility decline, evidence remains limited on perspectives and narratives taking shape in LMICs, including in sub-Saharan Africa (SSA), where the TFR remains higher than in most other regions of the world (7), and where FP has long been a development priority with broad stakeholder support. At the same time, distinct narratives are emerging, with some continuing to emphasize FP to address high fertility and unmet need for contraception, while others are increasingly focused on negative social and political impacts of fertility decline (8).

Stakeholders were keenly aware of fertility decline's global implications, but noted less attention to implications for safeguarding SRHR in their own policy and programming fora. Preliminary observations suggest that fertility decline concerns are not yet translating into widespread policy reversals in these countries.

However, respondents raised that these discussions are beginning to subtly influence policy at the national and subnational levels. For example, some cited fertility considerations entering national policy revision discussions; some identified sub-national regions where perceived too-low fertility is raising concern among local officials; and some described early shifts in priorities that could alter support for SRHR over time. Multiple stakeholders endorsed an optimal family size – a powerful message if adopted in policy.

Global and SSA stakeholders alike were acutely aware of examples from Asia and Latin America, where anti-rights actors are using fertility narratives to advance pronatalist arguments and policy. In SSA, a growing number of organized anti-rights groups oppose aspects of SRHR services, particularly abortion care, comprehensive sexuality education, and contraception access (9). Despite awareness of this, global SRHR leaders described their organizations as underprepared to counter these threats or proactively shape rights-protective fertility decline conversations, underscoring the need for resources to guide dialogue and address harmful narratives (8).

These observations do not establish a common regional trajectory, but they identify a preparedness gap that calls for anticipatory, coordinated, and locally tailored action by the SRHR community. The community should begin now to understand the fertility transitions in SSA and LMICs more broadly, learn the range and drivers of views and narratives, and protect rights by building locally relevant evidence, shaping rights-affirming communications, protecting services and financing, and safeguarding policy.

## Building locally relevant evidence

As fertility rate considerations begin to influence decision-making, strong data systems and reliable information are critical to designing SRHR-protective strategies. This includes context-specific demographic and health data to better understand national and sub-national fertility shifts.

Despite significant progress in recent decades, many LMICs continue to face gaps in the availability, completeness, and quality of data needed to monitor demographic change and related social indicators (10–12). Too often, population data systems and institutional capacity are under resourced, limiting the availability and use of quality data—a challenge exacerbated by recent reductions in global funding and limited research and development investments. Comprehensive SRHR data are essential to conversations on fertility, including geography-specific rates of unintended pregnancy and unmet need for contraception, which can remain high even where fertility rates are low, suggesting that reproductive needs and rights are not yet fully met.

When data are unavailable, inconsistent, or insufficiently disaggregated, they cannot support the tailored, context-specific responses that SRHR policy and programming require. Strengthening data is therefore not just a technical need, but a way to answer important questions, protect rights, and ground narratives in evidence. Communicating data nuances, and building the technical capacity to do so, are also critical for translating evidence into rights-based policy and programming (13–15).

Beyond population and health data, more qualitative information is needed on how narratives and anxieties evolve across contexts and the channels where discourse is developing, including among policy actors and the public.

Exploring these questions will require strategic partnership: trusted academic and research institutions and professional associations can generate and interpret evidence; statistical organizations and departments must be resourced to build and maintain data systems; civil society organizations (CSOs) can contribute community perspectives and promote accountability; and government partners can use quality data to promote effective policy and services. Together, these stakeholders can collaborate on locally led demographic, SRHR, and narrative research that identifies emerging risks and appropriate responses.

## Shaping rights-affirming narratives and communications

A rights-based communications approach should not seek or promote a particular level of fertility; it should enable people to achieve their reproductive intentions within the systems that support individuals and families. Our interviews and consultations suggest that fertility and demographic narratives are beginning to take on political dimensions, risking a shift in SRHR discussions away from evidence-based framing.

Data are critical in these conversations and must be clear, nuanced, and presented by trusted communicators. Because the anti-rights movement has a strong foothold in SSA, communication strategies should be coordinated with those countering broader anti-rights messages in the same communities. This is especially important, as the use of misinformation and disinformation by anti-rights actors can function as a policy tool to erode protections, a dynamic observed in similar settings where rights are under threat (16).

Research suggests support among policy makers, researchers, and advocates in SSA for integrating reproductive rights into population dynamics discussions (17), though messaging must balance rights-based communication with pragmatism to resonate broadly with stakeholders, including non-SRHR actors, such as finance and education ministries. Our initial consultations suggest that framing centered on healthy, supported families and reducing maternal mortality could bridge diverse priorities. Participants acknowledged needing more information on what messaging works and how to apply it, and raised the need for resources, including toolkits, to guide these conversations.

Engaging local, regional, and international media will be essential to protecting rights. Media translate technical information and political narratives into accurate, accessible reporting that informs public understanding and policy debate. Strengthening journalists' access to trusted experts, quality data, and training on fertility trends and SRHR can help reduce misinformation and ensure reporting reflects the best available evidence.

Educating the media is key to ensuring they effectively inform audiences with messages grounded in data and do not conflate different but related issues. For example, some audiences attribute declining fertility to rising infertility, even though global infertility prevalence has remained relatively stable and evidence does not support its contribution to the broader decline in fertility rates (18, 19).

Social media requires a different approach, as narratives spread rapidly and misleading information can gain traction faster than evidence. SRHR stakeholders should monitor emerging narratives, amplify trusted voices, and share accessible, evidence-based, and emotionally resonant content that shapes public discourse and counters misinformation. A strong, values-driven online presence can help keep rights and reproductive autonomy central in fertility decline discussions.

Communication approaches can be developed broadly, but must be tested and tailored locally, to prevailing perceptions and assumptions. For example, messages perceived as externally developed or insufficiently tied to local priorities and context may promote resistance.

Communications specialists, advocates, media partners, researchers, and CSOs can jointly test rights-affirming, locally relevant messages for policymakers, communities, and actors within and beyond the health sector.

## Protecting and strengthening health services and financing

Protecting SRHR in the context of fertility decline requires resilient health systems and financing that prevent demographic goals from driving service delivery and funding decisions in ways that restrict SRHR, compromise quality of care, or limit informed choice. These are pressure points where rights-eroding narratives could have concrete effects, such as pullback of SRHR services or budgets. Strengthening these foundations will help maintain access and quality and keep service delivery grounded in individual needs and rights rather than demographic targets. Continued investment in best practices will support this, including clinical training to promote unbiased, rights-based counseling and ensuring local availability of essential SRH supplies.

Funding allocation both signals and drives health priorities. At the country and regional levels, rapid decentralization of budget authority and decision-making has facilitated increasingly influential local governments (20, 21). Because health financing is determined at multiple levels, it is important to watch across governance structures for signs that fertility concerns are influencing budget priorities or threatening SRHR funding. Supporting civil society to track shifts and hold governments accountable for sustained, rights-based SRHR investment should be a priority, alongside coordinated action by ministries, technical assistance organizations, providers, local governments, and financing partners to protect systems and financing more broadly.

## Policy monitoring and safeguarding

As fertility concerns enter policy discussions, individual and family choices must be preserved (22). Doing so requires close policy tracking to ensure fertility decline alarm does not translate into policies that restrict SRHR. Governments, civil society, and development partners can proactively monitor the policy landscape for signals of rollback, including delaying, blocking or reversing supportive policy reforms, reduced SRHR budgets, institutional mergers that subsume SRHR into broader entities, and efforts to dilute SRHR policies. Advocates can also ensure that discussions of “optimal” fertility do not evolve into population control agendas. Framing fertility as a target can enable coercive, exclusionary, or nationalist policies, including those rooted in eugenics.

Where SRHR-supportive policies exist, governments can strengthen language to remove uncertainty and promote effective, rights-based implementation. Civil society has helped protect SRHR through interpreting SRHR policies, such as with Benin's expanded abortion law (23, 24). Although not a response to fertility decline, the Benin experience illustrates how governments and civil society can clarify policy before ambiguity is used to restrict access.

As with budgets, SRHR-protective policies and commitments must be defended and implemented. Years of advocacy have led to valuable gains, such as Tanzania's inclusion of family planning in universal health coverage (25). Governments, policy advocates, CSOs, and accountability actors should track emerging proposals, defend existing commitments (e.g., the ICPD PoA, Sustainable Development Goals, FP2030, Maputo Protocol), and respond early when fertility concerns risk imposing on rights.

## Discussion

Fertility decline's growing prominence in population and development debates presents an important opportunity for the SRHR community, but the implications are not uniform across settings. Fertility levels and trajectories vary widely, and in many LMICs remain relatively high. Our consultations suggest that concern about declining fertility has not yet translated into widespread policy or programmatic rollback. Rather, the clearest early signals are appearing in subnational policy discourse and narratives, and informal programmatic shifts in subnational regions where fertility has declined to relatively lower levels. These findings point to a need for preparedness before such concerns become more deeply embedded in policy and programming.

Global experience shows how quickly demographic concern can become linked to pronatalist, anti-rights, or crisis-based narratives. SRHR stakeholders therefore need to prepare across several interconnected fronts: generating locally relevant evidence, shaping rights-affirming narratives, protecting resilient services and financing, and monitoring policy for early signs of restriction or deprioritization. These priorities will vary by setting and should be locally led, but together they provide a common framework for anticipating and responding to emerging risks.

The SRHR community has previously countered opposition through coordinated advocacy and locally developed responses. In this case, we have a strategic window to proactively anticipate opposition rooted in fertility alarmism by shaping the narrative and preparing systems now. Governments, multilaterals, researchers, civil society, and funders all have a role in ensuring that responses to demographic change support people and families without compromising reproductive autonomy. This is our opportunity to shape that response before crisis-based narratives take hold.

## Data Availability

The data presented in this article are not publicly available because they were generated through informal stakeholder consultations conducted to inform program design and message development, rather than as part of a formal research study. The findings presented here are intended to illustrate emerging themes and perspectives and should not be interpreted as generalizable evidence. A brief is available at https://www.engenderhealth.org/at-a-crossroads-srhr-in-the-context-of-fertility-decline

## References

[1] United Nations Department of Economic and Social Affairs, Population Division. World Population Prospects 2024: Summary of Results, New York: United Nations (2024). Report No.: UN DESA/POP/2024/TR/NO. 9. Available online at: https://population.un.org/wpp/ (Accessed September 7, 2026).

[2] United Nations Population Fund. State of World Population 2023: 8 Billion Lives, Infinite Possibilities — the Case for Rights and Choices. New York: UNFPA (2023). Available online at: https://www.unfpa.org/sites/default/files/swop23/SWOP2023-ENGLISH-230329-web.pdf. (Accessed May 26, 2026)

[3] Boydell V Mori R Shahrook S Gietel-Basten S. Low fertility and fertility policies in the Asia-Pacific region. Glob Health Med. (2023) 5(5):271–7. 10.35772/ghm.2023.01058 37908516 PMC10615026

[4] Gietel-Basten S Rotkirch A Sobotka T. Changing the perspective on low birth rates: why simplistic solutions won't work. Br Med J. (2022) 379:e072670. 10.1136/bmj-2022-072670 PMC10915774 36379520

[5] United Nations. Programme of Action of the International Conference on Population and Development. New York: United Nations (1994).

[6] Note: These stakeholder engagements did not constitute formal research. They were conducted to inform message development and program design, and the findings presented are intended to illustrate key themes rather than provide generalizable evidence.

[7] Bhattacharjee NV Schumacher AE Aali A Abate YH Abbasgholizadeh R Abbasian M. Global fertility in 204 countries and territories, 1950–2021, with forecasts to 2100: a comprehensive demographic analysis for the global burden of disease study 2021. Lancet. (2024) 403(10440):2057–99. 10.1016/S0140-6736(24)00550-6 38521087 PMC11122687

[8] EngenderHealth. At a Crossroads: SRHR in the Context of Fertility Decline. Washington (DC): EngenderHealth (2026). Available online at: https://www.engenderhealth.org/at-a-crossroads-srhr-in-the-context-of-fertility-decline (Accessed September 7, 2026).

[9] Opondo E Maina J Munyasia N. Lessons from Kenya on sexual reproductive health and rights policy-making: the need to centre voices from Africa in global discourses. Sex Reprod Health Matters. (2024) 32(1):2409548. 10.1080/26410397.2024.2409548 39344802 PMC11488166

[10] Kashyap R. Has demography witnessed a data revolution? Promises and pitfalls of a changing data ecosystem.. Popul Stud (Camb). (2021) 75(Suppl 1):47–75. 10.1080/00324728.2021.1969031 34902280

[11] AbouZahr C De Savigny D Mikkelsen L Setel PW Lozano R Nichols E. Civil registration and vital statistics: progress in the data revolution for counting and accountability. Lancet. (2015) 386(10001):1373–85. 10.1016/S0140-6736(15)60173-8 25971224 PMC7753937

[12] Hoxha K Hung YW Irwin BR Grépin KA. Understanding the challenges associated with the use of data from routine health information systems in low- and middle-income countries: a systematic review. Health Inf Manag. (2022) 51(3):135–48. 10.1177/1833358320928729 32602368

[13] Hopkins J Narasimhan M Aujla M Silva R Mandil A. The importance of insufficient national data on sexual and reproductive health and rights in international databases. eClinicalMed. (2024) 70:102554. 10.1016/j.eclinm.2024.102554 PMC10963186 38533341

[14] Pomati M. Co-producing sexual and reproductive health and rights indicators-repurposing the consensual (poverty) approach for out-of-school girls. Child Indic Res. (2026) 19(3):1219–50. 10.1007/s12187-026-10337-y

[15] Starrs AM Ezeh AC Barker G Basu A Bertrand JT Blum R. Accelerate progress-sexual and reproductive health and rights for all: report of the guttmacher-lancet commission. Lancet. (2018) 391(10140):2642–92. 10.1016/S0140-6736(18)30293-9 29753597

[16] Purnat TD Wilhelm E Scales D Wardle C Bastien S Ganatra B. Impacts of sexual and reproductive health and rights misinformation in digital spaces on human rights protection and promotion: scoping review. JMIR Infodemiology. (2025) 5:e83747. 10.2196/83747 41468582 PMC12811040

[17] Delacroix C Owoo NS. Documenting the perspectives of sub-saharan African policy makers, researchers, and activists on the reproductive rights, population dynamics, and environmental sustainability nexus. World. (2023) 4(4):758–75. 10.3390/world4040048

[18] Mburu G Kiarie J Allotey P. Infertility services in the context of decreasing total fertility rates. Bull World Health Organ. (2026) 104(2):121–3. 10.2471/BLT.25.294210 41602493 PMC12834353

[19] Cox CM Thoma ME Tchangalova N Mburu G Bornstein MJ Johnson CL. Infertility prevalence and the methods of estimation from 1990 to 2021: a systematic review and meta-analysis. Hum Reprod Open. (2022) 2022(4):hoac051. 10.1093/hropen/hoac051 36483694 PMC9725182

[20] Resnick D. Subnational Democracy and Local Governance in Africa. Washington, DC: Brookings Institution; (2024). Available online at: https://www.brookings.edu/articles/subnational-democracy-and-local-governance-in-africa/. (Accessed May 12, 2026)

[21] Iddawela Y Lee N Rodríguez-Pose A. Quality of sub-national government and regional development in Africa. J Dev Stud. (2021) 57(8):1282–302. 10.1080/00220388.2021.1873286

[22] Tan J. Beyond fertility figures: towards reproductive rights and choices. Humanit Soc Sci Commun. (2024) 11:112. 10.1057/s41599-024-02608-2

[23] World Health Organization. Global Abortion Policies Database: Benin [Internet]. Geneva: World Health Organization (2023). Available online at: https://abortion-policies.srhr.org/country/benin?version=2023-06-16 (Accessed July 4, 2026)

[24] EngenderHealth. FY25 Impact Report: July 2024–June 2025. Washington (DC): EngenderHealth (2026). Available online at: https://e1.nmcdn.io/assets/engender/wp-content/uploads/2026/02/EngenderHealth_FY25_Impact-Report.pdf (Accessed July 20, 2026)

[25] Maricha J. Tanzania moves to include family planning in Universal Health Insurance. The Citizen. 2026 January 24 (Accessed 2026 July 19). Available online at: https://www.thecitizen.co.tz/tanzania/news/national/tanzania-moves-to-include-family-planning-in-universal-health-insurance-5335106

